# Pro-angiogenic effect of RANTES-loaded polysaccharide-based microparticles for a mouse ischemia therapy

**DOI:** 10.1038/s41598-017-13444-7

**Published:** 2017-10-16

**Authors:** N. Suffee, C. Le Visage, H. Hlawaty, R. Aid-Launais, V. Vanneaux, J. Larghero, O. Haddad, O. Oudar, N. Charnaux, A. Sutton

**Affiliations:** 1 INSERM, U1148, Laboratory for Vascular Translational Science, UFR SMBH, Université Paris 13, Sorbonne Paris Cité, Bobigny, France; 2 Inserm, UMR 1229, RMeS, Regenerative Medicine and Skeleton, Université de Nantes, ONIRIS, Nantes, France; 3 APHP, Hôpital Saint-Louis, Unité de Thérapie Cellulaire, Paris, France; Université Paris Diderot, Sorbonne Paris Cité, F-75475 Paris, France; 4 Inserm UMR1160 et CIC de Biothérapies, Institut Universitaire d’Hématologie, Hôpital Saint-Louis, Paris, France; 5 Laboratoire de Biochimie, Hôpital Jean Verdier, AP-HP, Bondy, France

## Abstract

Peripheral arterial disease results from the chronic obstruction of arteries leading to critical hindlimb ischemia. The aim was to develop a new therapeutic strategy of revascularization by using biodegradable and biocompatible polysaccharides-based microparticles (MP) to treat the mouse hindlimb ischemia. For this purpose, we deliver the pro-angiogenic chemokine Regulated upon Activation, Normal T-cell Expressed and Secreted (RANTES)/CCL5 in the mouse ischemic hindlimb, in solution or incorporated into polysaccharide-based microparticles. We demonstrate that RANTES-loaded microparticles improve the clinical score, induce the revascularization and the muscle regeneration in injured mice limb. To decipher the mechanisms underlying RANTES effects *in vivo*, we demonstrate that RANTES increases the spreading, the migration of human endothelial progenitor cells (EPC) and the formation of vascular network. The main receptors of RANTES *i*.*e*. CCR5, syndecan-4 and CD44 expressed at endothelial progenitor cell surface are involved in RANTES-induced *in vitro* biological effects on EPC. By using two RANTES mutants, [E66A]-RANTES with impaired ability to oligomerize, and [^44^AANA^47^]-RANTES mutated in the main RANTES-glycosaminoglycan binding site, we demonstrate that both chemokine oligomerization and binding site to glycosaminoglycans are essential for RANTES-induced angiogenesis *in vitro*. Herein we improved the muscle regeneration and revascularization after RANTES-loaded MP local injection in mice hindlimb ischemia.

## Introduction

The chronic obstruction of arteries is associated with a vessel wall resistance and a reduction of blood flow leading to critical limb ischemia. Actual treatments of peripheral arterial disease include balloon angioplasty, stenting or bypass graft surgery. However, novel therapies are currently being developed to promote the revascularization and the muscle regeneration.

Protein-based therapies using proangiogenic growth factors have been demonstrated to induce neovascularization in animal models of peripheral arterial diseases^1^. Vascular Endothelial Growth Factor (VEGF)-based gene therapy represents another option for the treatment of limb ischemia^1^. Up to date, basic Fibroblast Growth Factor (bFGF) constitutes the only protein-based therapy administered in clinical trials enrolling patients suffering from atherosclerotic peripheral arterial diseases^1^. The first clinical trial, published in 2000, identified the safety and the absence of significant adverse effects after intra-arterial infusion of bFGF^2^. The second clinical trial, published in 2001, was stopped prematurely because of proteinuria^3^. The third clinical trial, published in 2002, implicated intra-arterial bFGF injection and resulted in a 90-day change in peak walking time, ankle-brachial pressure index and demonstrated safety^4^. Another therapeutic axis for ischemia is represented by cell therapy. Indeed, the mobilization of endothelial progenitor cells (EPC) from bone marrow to peripheral blood, and their migration to ischemic sites may accelerate neovascularization^5^.

A randomized controlled study highlighted that transplantation of autologous bone marrow-derived mononuclear cells could reduce the amputation associated with limb ischemia^6^. Chemokines or chemokine receptors such as CXCR3 could be of importance in cell therapy approaches since they are known to participate in arteriogenesis, angiogenesis and muscle regeneration^7^. Monocyte Chemotactic Protein-1 (MCP-1)/CCL2 is a chemokine involved in early inflammation and muscle regeneration following hindlimb ischemia in mice^7^. The effect of Stromal Derived Factor-1 (SDF-1)/CXCL12 on mobilization and migration of EPC from the bone marrow to the ischemic site has been reported^8^. The CC-chemokine Regulated upon Activation, Normal T-cell Expressed and Secreted (RANTES)/CCL5 is both a T cell chemoattractant and an immune-regulatory molecule. We previously described, on various cell types, that RANTES binds to its specific G protein-coupled receptors (GPCR) CCR1, CCR3 and CCR5 and to proteoglycans such as syndecan (SDC)-1, SDC-4 and CD44^9–12^. The binding of RANTES to its specific GPCR or to heparan sulfate proteoglycans resulted in the migration of human hepatoma cells or endothelial cells^11–13^. RANTES has been detected in plasma samples of patients with cardiovascular diseases^14^ and is involved in cardiac inflammatory disorders after organ transplantation or arterial injury^15^.

The expression of RANTES receptors, CCR1 and CCR5, on various cell types implicated in atherosclerosis further illustrates their role in this disease^15^. It has been previously demonstrated that RANTES may induce the production of VEGF, thus leading to angiogenesis in various disease animal models^12,16–18^. Our hypothesis is that RANTES may promote chemokine-induced angiogenesis and accelerate the tissue regeneration after hindlimb ischemia. The beneficial effects of proangiogenic chemokines in the treatment of experimental hindlimb ischemia relate to their capacity to induce the homing of proangiogenic bone marrow derived cells. However, the use of a chemokine is limited by its sensitivity to the intense proteolytic activity found in inflammatory injured tissues and its short half-life, *in vivo* ^19^. A biomaterial approach that could control and sustain the local prolonged release of RANTES may constitute therapeutic tools optimizing its effects on stem cell homing to the injured sites and neovascularization^20^. Our laboratory previously developed polysaccharide-based hydrogel architectures and three-dimensional scaffolds suitable for endothelial cell therapy, growth factor release applications and neo-vascularization in mice^21–25^.

Hence, the aim of this study was to develop a new therapeutic angiogenic approach by delivering RANTES, incorporated into polysaccharide-based microparticles, in the mice ischemic hindlimb. We demonstrated that local administration of this chemokine following the ischemic injury could promote the early reparative events preceding the neovessel formation, muscle regeneration and the restoration of blood flow.

## Results

### Improvement of clinical score after loaded-microparticle injection

**Figure 1:**
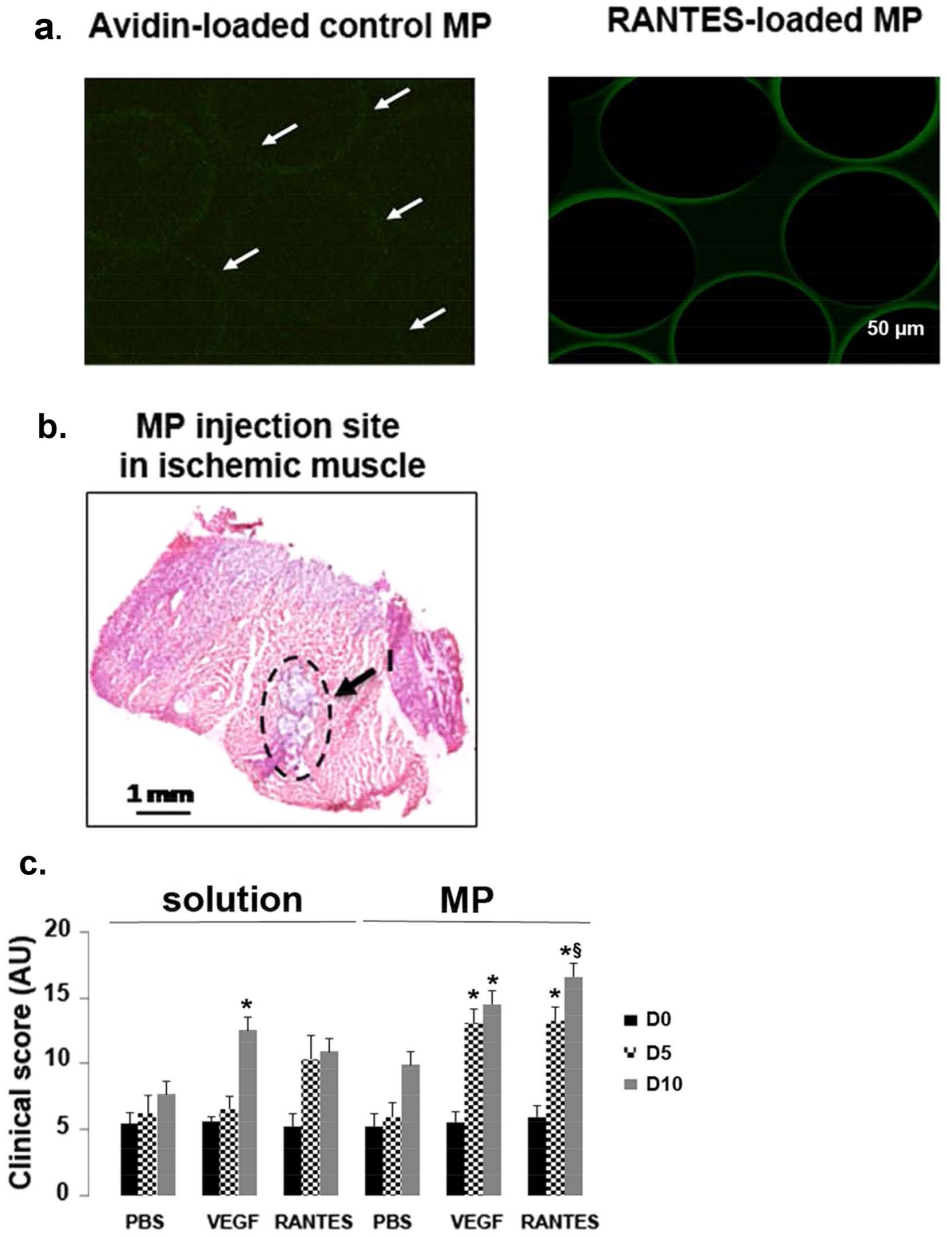
Improvement of clinical score after RANTES-loaded microparticle injection. **(a)** Representative confocal microscopy image of pullulan/dextran-based microparticles (MP) incubated with avidin-FITC alone (Avidin-loaded control MP) or with 10 nM biotinylated-RANTES followed by avidin-FITC to assess RANTES localization. Scale Bar: 50 µm. **(b)** 10 nM biotinylated RANTES-loaded MP (15 µg/mL) were injected intramuscularly in 6-week old male BALB/c white mice into their ischemic limb. At day 0, RANTES-loaded MP were identified in the injection area (I), as indicated with arrows on hematoxylin-eosin stained sections of the ischemic tissue, observed with optical microscopy. Bar: 1 mm. **(c)** Histograms represent clinical scores calculated as the sum of the grade obtained for Tarlov score, ischemic score and modified ischemic score at days 0, 5 and 10 after hindlimb ischemia. The effect of two different ways of treatment administration by injection of PBS, VEGF (2 nM) or RANTES (10 nM) solution or by PBS-, VEGF- or RANTES-loaded MP intramuscular injection in ischemic limb was evaluated,*P < 0.05 *versus* PBS solution at day 10.

Chemically cross-linked hydrogels based on biodegradable polysaccharides pullulan and dextran developed by our laboratory can be loaded with peptides and have been shown to favor the delivery of proangiogenic factors^25^. As compared to negative control microparticles (MP) alone incubated with avidin-FITC where no green fluorescent was observed, localization of biotinylated RANTES within the MP appeared homogeneously and weak inside the MP, but stronger on their surface, as evidenced by the green fluorescence ring (Fig. 1a). After MP incubation in PBS for 1, 7 or 14 days, RANTES was released at 2.5 ± 0.004 pg/mL (n = 3). Moreover, MP biodegradation induced by dextranase and pullulanase demonstrated that 6 ± 0.03 pg/mL of RANTES could be released from the digested MP (n = 3, Supplementary Fig. 1a). The localization of biotinylated RANTES was assessed after RANTES revelation with streptavidin-FITC labeling and by immunohistochemistry at 5 and 10 day post-injection in muscle tissue sections. We observed expression of RANTES around beads and in tissue periphery at 5 day post-injection. At 10 day post-injection, RANTES was observed as a diffuse signal in the tissue, confirming that an actual release took place (Supplementary Fig. 1b).

Injection of PBS, VEGF or RANTES solutions or injection of MP loaded with PBS, VEGF or RANTES was performed directly into the muscle immediately after the femoral artery ligature in mice. The MP were easily identified on tissue sections using optical microscopy (Fig. 1b). The clinical scores of muscle regeneration after hindlimb ischemia were calculated at day 0, 5 and 10. The injection of VEGF solution increased significantly the mice clinical score at day 10, but not at day 5, as compared to PBS or RANTES solution injection (Fig. 1c, n = 5, *P* < 0.05). It is to note that the clinical score calculated at day 5 or 10 after RANTES solution injection was quite similar but not significantly different of PBS solution injection.

The injection of RANTES- or VEGF-loaded MP increased at the same extent the clinical score at day 5 or 10, suggesting that they both improved the animal behaviour (Fig. 1c, n = 5, *P* < 0.05). It is worth noting that negative control MP had no significant effect. We then sacrificed the animals at day 10 which corresponds to the period required to observe endothelial progenitor cell (EPC) recruitment to injured muscles and their regeneration^26,27^.

Then we wanted to analyze if the increased clinical score associated with healthier animal behavior after RANTES-loaded MP treatment was associated with muscle regeneration.

### Muscle regeneration of hindlimb ischemia after loaded-microparticle injection

**Figure 2:**
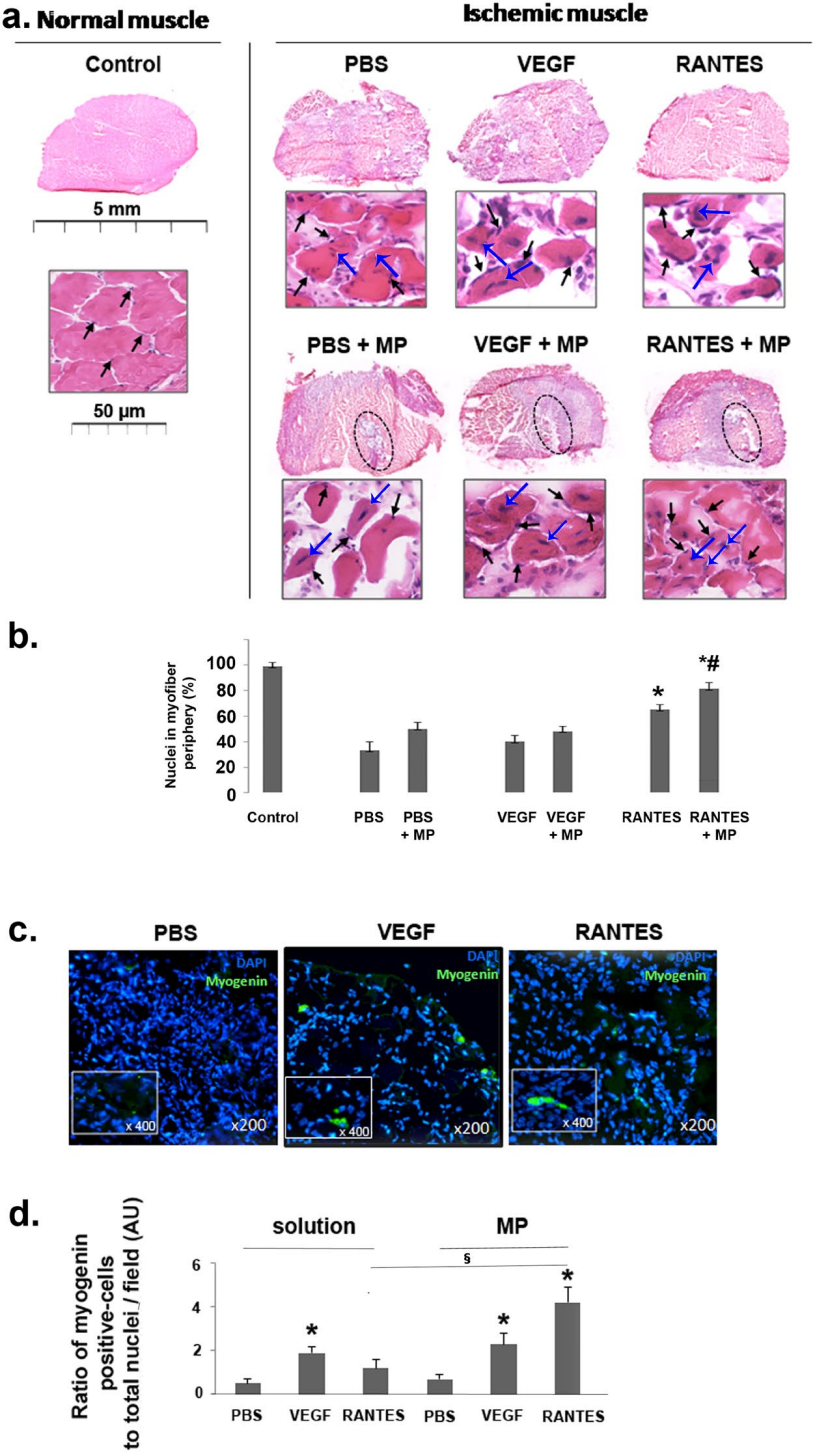
Induction of muscle regeneration after hindlimb ischemia. **(a)** Regeneration process after ischemia was analyzed in the muscles cross-sections stained with haematoxylin-eosin (upper panels, scale bar = 5 mm and lower panels, scale bar = 50 μm) after treatment with PBS, VEGF and RANTES solution or after treatment with PBS-, VEGF- or RANTES-loaded MP. The zone of MP injection was circled. Black arrows indicate the nuclear localization in the periphery of the mature muscle fibers. Blue arrows indicate the nuclear localization in the central part of the activated immature muscle fibers. Images were chosen as representative of total cross-section analysis of 5 muscles per group. **(b)** Histogram represents the percentage of nuclei located in the periphery of myofibers in muscle cryosection of mice treated with PBS, VEGF (2 nM) or RANTES (10 nM) solutions or with MP-loaded with PBS, VEGF (2 mM) or RANTES (10 nM),*P < 0.05 *versus* PBS, ^§^ *P* < 0.05 RANTES-loaded MP *versus* RANTES solution. **(c)** Ten days after hindlimb ischemia, the MP-injected tissue cryosections were immunolabeled with anti-myogenin D antibody revealed with Alexa Fluor 488 (green) secondary antibodies whereas the nuclei were stained with DAPI (blue). Magnification: × 200, inset × 400. **(d)** Histogram represents the ratio of myogenin D positive cells per total nuclei in cryosection field of mice treated with PBS, VEGF (2 nM) or RANTES (10 nM) solutions or with MP-loaded with PBS, VEGF (2 nM) or RANTES (10 nM),*P < 0.05 *versus* PBS, ^§^ *P* < 0.05 RANTES-loaded MP *versus* RANTES solution.

The location of nuclei in the muscle myofibers is relative to the evolution of ischemia or muscle regeneration process^28,29^. In a healthy muscle, the nuclei are present at the periphery of mature muscle myofibers (Fig. 2a left panel, black arrows). Muscle injury leads to muscle damage characterized by a loss of myofiber organization. Muscle regeneration and maturation is then characterized by migration of nuclei of myofibers from the center (associated with immature myofibers) to the periphery (associated with mature myofibers)^30,31^. After PBS solution or negative control MP injection in ischemic muscle, the nuclei appear predominantly at the center of the myofibers. Treatment with VEGF or RANTES solutions or VEGF-loaded MP led to a partial migration of nuclei from the center (Fig. 2a top and bottom middle panels, blue arrows) to the periphery part of myofibers (Fig. 2a top and bottom middle panels, black arrows). Evident myofiber maturation is only observed after RANTES-loaded MP treatment with the numerous nuclei localized at the periphery of myofibers (Fig. 2a bottom and right panel, black arrows).

A quantitative analysis of the number of myofibers with peripheral and central nuclei indicated that the percentage of peripheral nuclei, marker of myofiber maturity, is significantly higher after RANTES injection. It is to note that the delivery with MP increased significantly RANTES effect as compared to the injection of RANTES in solution (Fig. 2b). Myogenin D is considered as a biomarker of muscle functionality^32^. Therefore, we assessed the expression of myogenin D by immunohistochemistry assay (Fig. 2c).The ratio of myogenin D positive cells normalized to the total number of cells, evidenced by DAPI nuclei staining, was significantly increased by 2 fold after the injection of VEGF solution or VEGF-loaded MP. In contrast, this ratio was unchanged after RANTES solution treatment but was significantly increased by 4-fold after injection of RANTES-loaded MP (Fig. 2d, n = 5, *P* < 0.05). The aim of the next step was to evaluate if muscle regeneration is associated with tissue revascularization.

### Revascularization of hindlimb ischemia after loaded-microparticle injection

**Figure 3:**
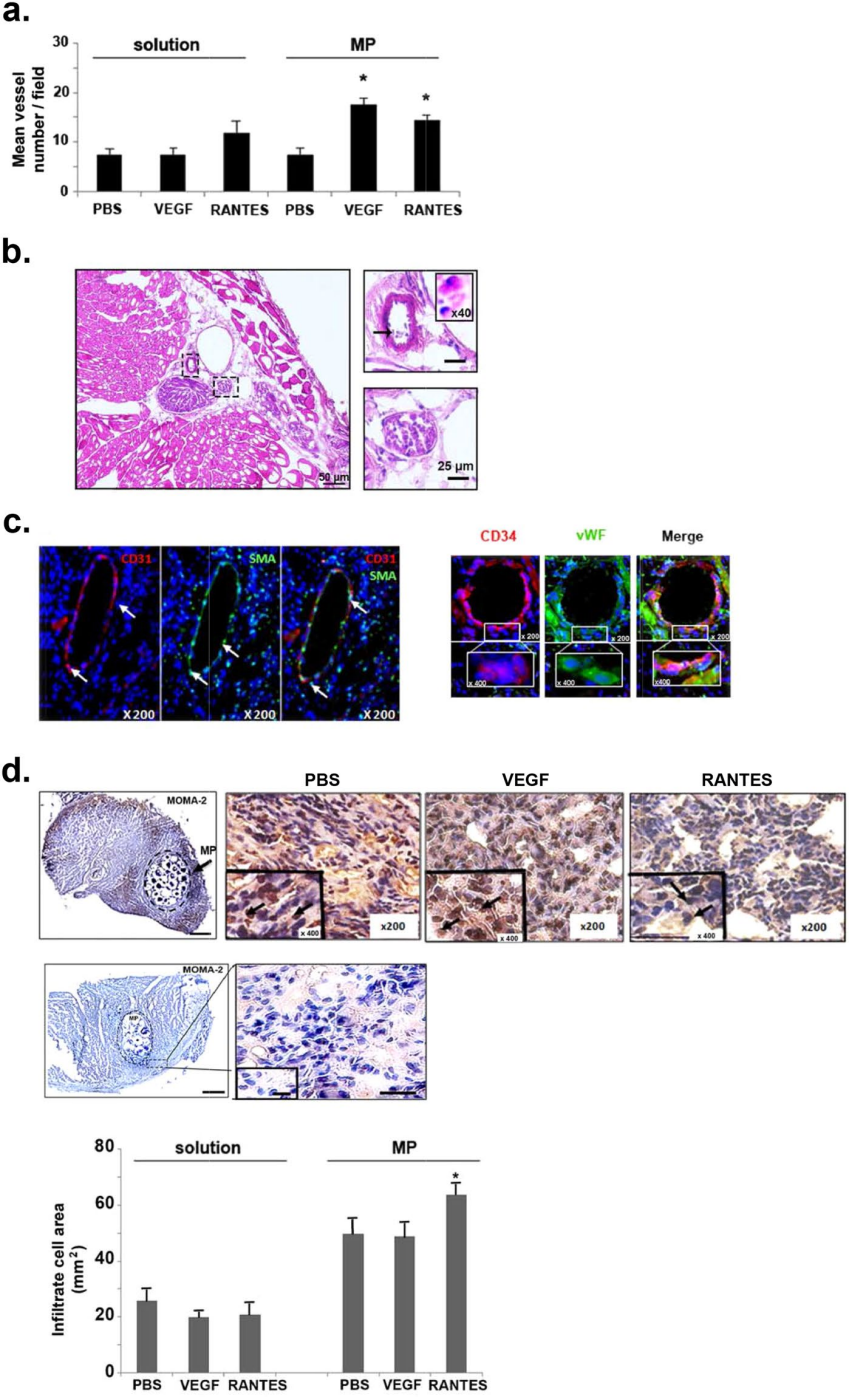
Induction of revascularization after hindlimb ischemia. (**a**) Quantification of microvessel density on hematoxylin-eosin frozen sections. Histograms represent mean vessel number per field of hematoxylin-eosin cryosections counted by two independent observers, 10 days after hindlimb ischemia. Results are expressed as mean ± SEM. *P < 0.05 *versus* PBS. **(b)** The presence of inflammatory cells such as polynuclear neutrophils (indicated with black arrow) and erythrocytes in the lumen of microvessels revealed by a hematoxylin-eosin staining indicate the functionality of blood microvessels. Bar = 10 µm, magnification inset × 400. **(c)** *Left panel:* Endothelial cells were identified by immunohistochemistry with anti-CD31 antibody revealed with Alexa fluor 555-secondary antibody (red) and vascular smooth muscle cells were immunolabeled with anti-smooth muscle actin (SMA) antibody revealed with Alexa fluor 488-secondary antibody (green). Nuclei were stained with DAPI (blue). Magnification: × 200. *Right panel:* Cells derived from endothelial progenitor cells were identified in the intima of microvessels in samples treated with MP loaded RANTES (10 nM) by immunohistochemistry with anti-CD34 antibody revealed with Alexa fluor 555-secondary antibody (red) and with anti-vWF antibodies revealed with Alexa fluor 488-secondary antibody (green). Nuclei were stained with DAPI (blue). Magnification: × 200, inset × 400. **(d)** *Upper panel:* Monocytes-macrophages were identified with anti-MOMA-2 antibody followed by HRP-labeled secondary antibodies and DAB with nuclei stained in blue with hemalum indicated with black arrows in high insert view. Bar: 1 mm (*Upper left panel*), Magnification: × 200, inset × 400 (*Upper right panel*). *Middle panel*: Negative control of monocytes-macrophages immunostaining. Slides were incubated with specific isotype instead of anti-MOMA-2 antibody, then with HRP-labeled secondary antibodies and DAB, and nuclei were stained in blue with hemalum. Bar: 1 mm (*Middle left panel*), Magnification: × 200, inset × 400 (*Middle right panel*). *Lower panel*: Histogram represents infiltrate cell area evidenced by anti-MOMA-2 immunostaining. Results are expressed as mean ± SEM of the ratio between infiltrated cells surface area and the total surface area of the section.

Muscle ischemia results from a tissue devascularization induced by surgery and ligature procedures. Thus, myogenesis and angiogenesis are the main processes involved in muscle regeneration^33,34^. Quantification of the blood microvessel density was evaluated on hematoxylin-eosin stained muscle cross sections of mice sacrificed at day 10. Only VEGF- or RANTES-loaded MP significantly increased by 2.6-fold or 2.3-fold, respectively, the number of blood vessel per field, whereas the other treatments did not affect it significantly (Fig. 3a). The injection of either RANTES solution led to similar microvessel density, without reaching a statistical significance *versus* PBS solution (Fig. 3a).The neo-formed microvessels at the periphery of the injection site, obtained after injection of PBS-, VEGF- or RANTES-loaded MP were polymorph, mainly small and thin with regular shape. Moreover, the presence of erythrocytes or polynuclear neutrophils within almost all capillaries on hematoxylin-eosin stained cross sections evidenced the functionality of the neo-formed microvessels (Fig. 3b). These neo-formed microvessels were composed of endothelial cells (EC) as assessed by CD31 immunolabelling and of vascular smooth muscle cells (VSMC) as revealed by alpha smooth muscle actin (SMA) immunolabelling (Fig. 3c left panel). Expression of these markers suggests the formation of mature blood neovessels with an intima layer containing EC and a media layer containing VSMC. The muscle regeneration induced after RANTES-loaded MP treatment may be related to a direct pro-angiogenic effect on mature endothelial cells^12^, or a recruitment of EPC as it was previously described for the chemokine SDF-1^33^. The presence of cells derived from EPC in the zone of revascularization was evidenced by immunolabelling of their membrane with the specific markers. Undifferentiated progenitor cells can be identified by CD34 expression^34,35^.

Hence, at the periphery of microvessels some cells expressed CD34, suggesting that these cells kept a membrane marker of undifferentiated progenitor cells. These cells also expressed endothelial-specific markers such as CD31 (data not shown) and von Willebrandt factor (vWF, Fig. 3c right panel), suggesting an initiation of the differentiation process into EPC. To evaluate the cellular processes involved in RANTES-induced revascularization, we therefore investigated the *in vitro* RANTES-induced EPC migration and a two-dimensional (2D) vascular network formation assay.

The chemokine-induced angiogenesis may result from growth factor expression or leukocyte infiltration^32^. The inflammatory cell infiltration was assessed by monocytes/macrophages immunolabelling using anti-MOMA-2 antibodies (Fig. 3d, upper left panel). Infiltrate cell area assessed on hematoxylin-eosin cross section is localized in an area of less visible myofibers. Infiltrate cell area is increased by a 2-fold factor after administration of PBS-loaded MP compared to PBS solution (Fig. 3d, upper right panel). Infiltrate cell area was significantly decreased by 23 ± 1% after VEGF (20 ± 1.4 mm²) or by 19 ± 7% after RANTES (21 ± 3 mm²) solution treatment as compared to the PBS control (26 ± 4 mm², Fig. 3d upper right panel, n = 5, *P* < 0.05). In contrast, the infiltrate cell area was similar to PBS-loaded MP (50 ± 6 mm²), in VEGF-loaded MP treatment (49 ± 5 mm²) and it was significantly increased by 128 ± 4% (64 ± 4 mm²) after a RANTES-loaded MP treatment (Fig. 3d upper right panel, n = 5, *P* < 0.05). The inflammatory cell infiltration evidenced by immunolabelling using anti-MOMA-2 antibodies was more pronounced on muscle after RANTES-loaded MP treatment (Fig. 3d, lower panel, n = 5, *P* < 0.05).

The identification of CD34 + CD31 + vWF + cells in the tissue of RANTES-loaded MP treated mice led us to investigate the RANTES-induced biological effects on EPC *in vitro*. Among these effects, we focused on mechanisms involved in angiogenesis *i*.*e* EPC spreading, migration and sprouting assays.

### RANTES-induced EPC spreading, migration and sprouting

**Figure 4:**
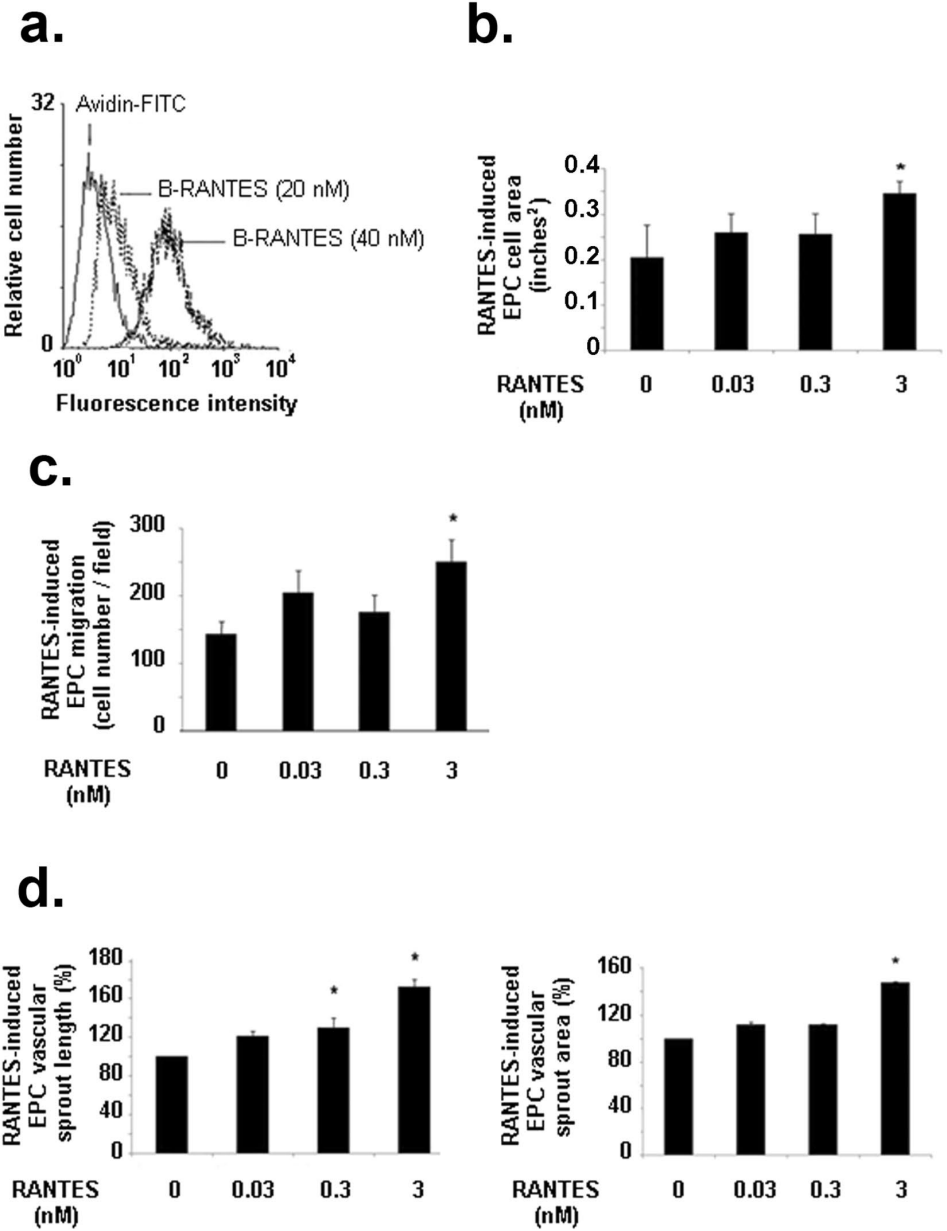
Biological effects induced by RANTES on EPC. **(a)** RANTES binds to EPC in a dose-dependent manner. Human EPC were incubated with 20 or 40 nM biotinylated RANTES (B-RANTES) and the binding were analysed by flow cytometer with avidin-FITC. Reactivity was compared to avidin-FITC alone. Data shown are representative of three independent experiments. (**b)** RANTES at 3 nM induced EPC spreading on fibronectin layer. Results are expressed as mean ± SEM of EPC area, expressed in square-inches, measured by field for three independent experiments. *P < 0.05 *versus* untreated cells. **(c)** RANTES at 3 nM induced EPC migration in a modified Boyden chamber model. Results are expressed as mean ± SEM of EPC counted by field for three independent experiments. *P < 0.05 *versus* untreated cells. (**d)** RANTES at 3 nM induces EPC vascular sprout area (*left panel*) and vascular sprout length (*right panel*) and in a 2D angiogenesis assay on Matrigel. The length and area of vascular sprouts formed by untreated EPC was arbitrary set to 100%. The results for cells treated with RANTES were expressed as a percentage of control cells. *P < 0.05 *versus* untreated cells.

Biotinylated RANTES binds to EPC in a dose dependent manner (Fig. 4a). Only RANTES at 3 nM significantly increased EPC spreading by 69 ± 9%, conversely to lower concentrations (0.03 or 0.3 nM) (Fig. 4b, n = 3, *P* < 0.05). Similarly, RANTES at 3 nM increased significantly EPC migration by 72 ± 22%, as compared to untreated cells, conversely to lower concentrations (Fig. 4c, n = 3, *P* < 0.05). A dose-dependent effect of RANTES on the length of vascular sprout formed in a 2D-angiogenesis assay was evidenced, as 0.3 nM or 3 nM RANTES increased it by 31 ± 1% or 72 ± 8%, respectively (Fig. 4d, n = 3, *P* < 0.05). In addition, only 3 nM RANTES significantly increased by 48 ± 1% the area of the formed vascular sprout, as compared to untreated cells (Fig. 4d, n = 3, *P* < 0.05).

Considering the biological effects of RANTES on EPC, and the role of chemokines binding to glycosaminoglycans or oligomerization in their induced biological effects, we evaluated the effects of RANTES mutants on EPC migration and sprouting.

### RANTES-mediated effects depend on its oligomerization and its binding to glycosaminoglycans

**Figure 5:**
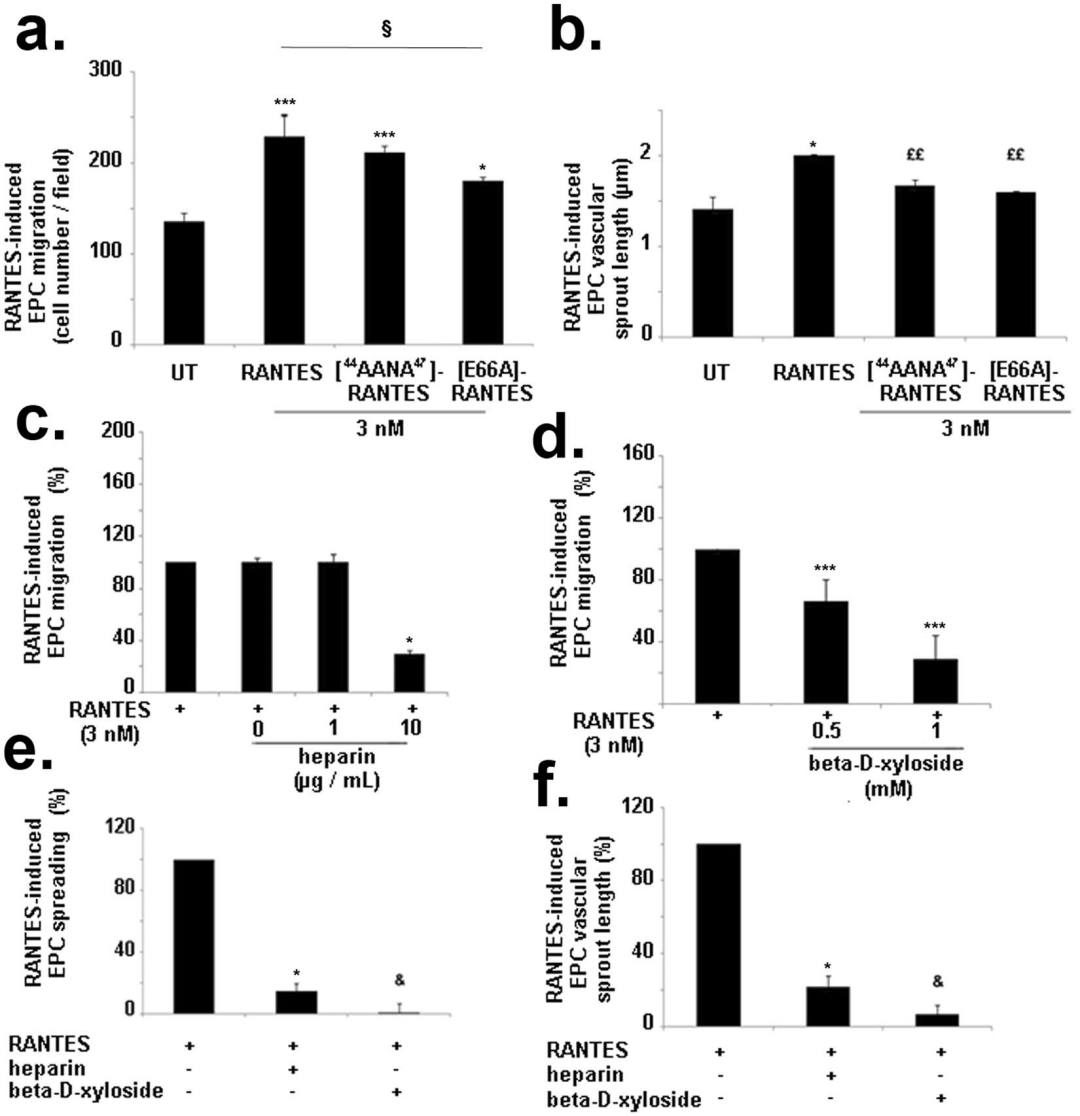
Glycosaminoglycans are involved in RANTES-induced biological effects. **(a,b)** EPC were incubated with RANTES or [^44^AANA^47^]-RANTES or [E66A]-RANTES mutants (each at 3 nM). **(a)** EPC migration was assessed by Transwell assay. Results are expressed as mean ± SEM of EPC counted by field for three independent experiments. *P < 0.05 or ***P < 0.001 *versus* untreated cells (UT). ^§^ *P* < 0.05 *versus* RANTES. **(b)** EPC vascular sprout length was assessed in a 2D angiogenesis assay. Histogram represents cells treated with RANTES or [^44^AANA^47^]-RANTES or [E66A]-RANTES (each at 3 nM). *P < 0.05 *versus* untreated cells (UT). ^££^ *P* < 0.01 *versus* RANTES. **(c)** EPC migration induced by RANTES in absence of heparin was arbitrary set to 100%. The results of cells stimulated by 3 nM RANTES preincubated with a range of heparin concentrations (at 0.1, 1, 10 µg/mL) were expressed as a percentage of RANTES alone. *P < 0.05 *versus* cells stimulated by RANTES in the absence of heparin. **(d)** RANTES (3 nM) induced migration of EPC pre-treated with beta-D-xyloside (at 0.5 or 1 mM). The migration of untreated EPC towards RANTES was arbitrary set to100%. The results of cells treated with beta-D-xyloside and chemoattracted by RANTES were expressed as a percentage of untreated cells chemoattracted by RANTES. ***P < 0.001 *versus* untreated cells (UT) chemoattracted by RANTES. **(e,f)** EPC area induced by RANTES (3 nM) was assessed after a cell spreading assay **(e)** and the length of vascular sprouts formed by EPC was assessed after a 2D angiogenesis assay **(f)**. *P < 0.05, cells stimulated by RANTES pre-incubated with 10 µg/mL heparin *versus* cells stimulated by RANTES in absence of a preincubation with heparin. ^&^ *P* < 0.05 cells treated with 1 mM beta-D-xyloside and chemoattracted by RANTES *versus* untreated cells chemoattracted by RANTES.

The mutation [E66A]-RANTES affects the oligomerization status leading to a dimeric form of RANTES. [E66A]-RANTES increased by 31 ± 5% the EPC migration compared to untreated cells (Fig. 5a, n = 3, *P* < 0.05). Thus, [E66A]-RANTES is 22 ± 5% less efficient than RANTES to induce EPC migration (Fig. 5a, n = 3, *P* < 0.001). [E66A]-RANTES increased the length of the formed vascular sprout by 15 ± 4% compared to untreated cells (Fig. 5b, n = 3, *P* < 0.01). Thus, [E66A]-RANTES was significantly 4.8 fold less efficient than RANTES to increase the length of capillaries formed (Fig. 5b, n = 3, *P* < 0.05). To assess the role of RANTES binding to GAG, the experiments were performed either with the [^44^AANA^47^]-RANTES, that is deficient in glycosaminoglycan binding sites, or with the chemokine preincubated with low molecular weight heparin (LMWH), or after the cell pretreatment with a beta-D-xyloside, an inhibitor of GAG biosynthesis. The mutant [^44^AANA^47^]-RANTES increased EPC chemotaxis by 49 ± 6% and vascular sprout length by 21 ± 4%, relative to untreated cells (UT) (Fig. 5a and b, n = 3, *P* < 0.01 and *P* < 0.001, respectively). Thus, [^44^AANA^47^]-RANTES is 11 ± 4% and 2.9 fold less efficient than RANTES to induce EPC migration or vascular sprout formation respectively (Fig. 5b, n = 3, *P* < 0.01). These results demonstrated that RANTES oligomerization is essential in EPC migration and vascular sprout formation; whereas RANTES binding to GAG is necessary only for EPC vascular sprout formation.

The preincubation of RANTES with 10 µg/mL LMWH reduced the cell migration by 70 ± 3% whereas lower doses did not affect it (Fig. 5c, n = 3, *P* < 0.05). Interestingly, upon 3 nM RANTES treatment, heparin reduced EPC spreading by 85 ± 4% (Fig. 5e, n = 3, *P* < 0.05), and decreased vascular sprout length by 78 ± 6% (Fig. 5f, n = 3, *P* < 0.05). It is to note that heparin, in absence of RANTES treatment, did not affect EPC spreading, migration and sprouting. The cell pretreatment with two different non-cytotoxic concentrations of beta-D-xyloside significantly reduced in a dose-dependent manner the migration of EPC, by 44 ± 3% for a 0.5 mM beta-D-xyloside concentration, or by 71 ± 12% for a 1 mM beta-D-xyloside concentration induced with 3 nM RANTES (Fig. 5d, n = 3, *P* < 0.001). Without RANTES, cell pretreatment with 1 mM beta-D-xyloside did not affect cell migration but increased vascular sprout length by 63 ± 10% as compared to basal condition (n = 3, data not shown). Human EPC spreading was abolished after cell pretreatment with 1 mM beta-D-xyloside (Fig. 5e, n = 3, *P* < 0.05).

The length of vascular sprout formed in a 2D angiogenesis assay decreased by 95 ± 5% after cell pretreatment with 1 mM beta-D-xyloside (Fig. 5f, n = 3, *P* < 0.05). According to these results, the cellular effect of RANTES may rely on its binding to glycosaminoglycans, but also to specific G protein-coupled receptors (GPCR). Hence the binding of RANTES to its or GPCR or to glycosaminoglycans mostly carried by membrane proteoglycans such as SDC-1, SDC-4 or CD44, may constitute the first step of RANTES cell signalling leading to these observed proangiogenic cellular effects.

### Involvement of RANTES (co)-receptors in RANTES-induced angiogenic effects

**Figure 6:**
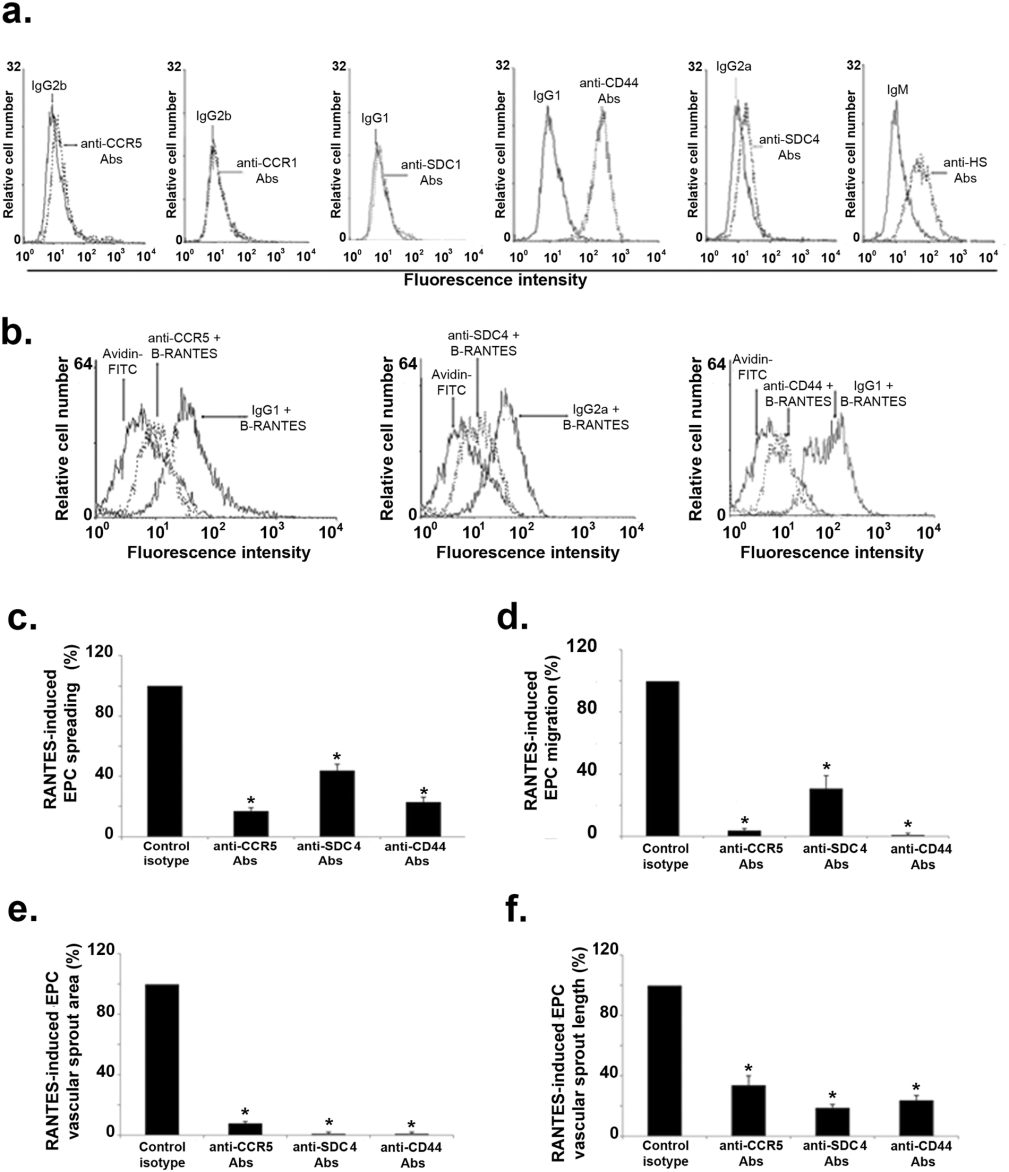
Expression of G protein-coupled receptors and proteoglycans on EPC. **(a)** Flow cytometry analysis of cells incubated with anti-CCR1, anti-CCR5, anti-SDC-1, anti-SDC-4, anti-CD44, anti-heparan sulfate (HS) antibodies or their respective isotypes and with Alexa Fluor-488 labeled secondary antibodies. Data shown are representative of three independent experiments. (**b**) Inhibition of biotinylated RANTES (40 nM) binding by preincubation of EPC with anti-CCR5, anti-SDC-4, anti-CD44 antibodies or their respective isotypes. Binding of B-RANTES was analyzed by flow cytometer with avidin-FITC control condition. Reactivity was compared to avidin-FITC. **(c)** The effect of G protein-coupled receptors and proteoglycans in RANTES-mediated cell spreading **(c)**, migration **(d)** and 2D angiogenesis assays **(e, f)** was assessed after preincubation of EPC with anti-CCR5, anti-SDC-4, anti-CD44 antibodies or their respective isotypes. RANTES-induced EPC spreading, migration, vascular sprout area and length of EPC pre-incubated with the isotype were arbitrary set to 100%. *P < 0.05 *versus* cells preincubated with specific isotypes.

The expression of RANTES specific GPCR, CCR1 and CCR5, and of proteoglycans, SDC-1, SDC-4, CD44, were analyzed on EPC membrane using flow cytometry. The membrane expression of CCR1 or SDC-1 was undetectable; whereas CCR5, SDC-4, CD44 and heparan sulfate (HS) chains were expressed at EPC cell surface (Fig. 6a). Cell incubation with anti-CCR5, anti-CD44 or anti-SDC-4 antibodies decreased RANTES binding to EPC by 92 ± 1%, 92 ± 4% or 89 ± 4 respectively, as compared to their respective isotypes (Fig. 6b, n = 3, *P* < 0.05).

The EPC preincubation with anti-CCR5, anti-SDC-4 or anti-CD44 antibodies significantly reduced the RANTES-induced cell spreading by 83 ± 4%, 56 ± 5%, 77 ± 12%, respectively (Fig. 6c, n = 3, *P* < 0.05).

The RANTES-induced migration of EPC was significantly reduced by 96 ± 3%, 69 ± 19%, and abolished after cell preincubation with anti-CCR5, anti-SDC-4 or anti-CD44 antibodies, respectively (Fig. 6d, n = 3, *P* < 0.05). The RANTES-induced increase of vascular sprout area was significantly reduced by 92 ± 2% after EPC preincubation with anti-CCR5, and abolished after cell preincubation with anti-SDC-4 or anti-CD44 antibodies (Fig. 6e, n = 3, *P* < 0.05).

The RANTES-induced raising of vascular sprout length was reduced by 66 ± 11%, 81 ± 10%, 76 ± 8% after cell preincubation with anti-CCR5, anti-SDC-4 or anti-CD44 antibodies, respectively (Fig. 6f, n = 3, *P* < 0.05).

Altogether, our results demonstrate that EPC express membrane receptors and proteoglycans able to bind RANTES. RANTES binding to these cells leads to pro-angiogenic cellular effects such as EPC migration and vascular sprouting.

## Discussion

Patients with critical limb ischemia develop a blood monocytosis that may contribute to the tissue repair process, notably by the secretion of growth factors or chemokines^7^. Angiogenesis is important for neovascularization of ischemic tissues and protein-based or gene-based therapy using angiogenic factors such as bFGF or VEGF, is under investigation in clinical practice^1^. However, these strategies appear to be not very convincing in patients with myocardial ischemia^7^. The rapid diffusion of an angiogenic protein and its degradation by inflammation-related proteolytic enzymes may reduce its local concentration and its angiogenic effect. Therefore, the use of a biomaterial to deliver locally an angiogenic factor such as VEGF, with a controlled release, at the injured site, can be considered as an advanced and challenging therapeutic strategy^36,37^. Incorporation of the chemokine SDF-1 into a heparin-based hydrogel was shown to induce chemotaxis *in vitro* and potentiate EPC enrichment subcutaneously in mice^20^. It has been demonstrated that accumulation of infused endothelial progenitors can be enriched using biomaterial-based delivery of VEGF and SDF-1^38^. Such an added effect of RANTES-loaded MP and VEGF-loaded MP co-delivery in our mice hindlimb ischemia model needs to be investigated. However, as a competition in the ionic and electrostatic association between RANTES or VEGF and the pullulan-dextran scaffold may occur, some preliminary experiments are required to better define the respective concentrations of RANTES-loaded MP and VEGF-loaded MP inducing angiogenesis without adverse effects. The respective optimal amount of the chemokine and of the growth factor to enable the most efficient neo-revascularization is not well defined, specifically *in vivo*. Among chemokines released in ischemic lesions, RANTES is secreted by many cell types such as macrophages, activated T cells, platelets, endothelial cells and smooth muscle cells. Through its binding to its receptors CCR1, CCR3 or CCR5, this chemokine is known for its role in the homing and activation of inflammatory cells. The pro-angiogenic role of RANTES in cancer has been suggested by its ability to increase vascularity^39^. RANTES pro-angiogenic effects have been evoked previously but controversy still remains^39^. Indeed, Barcelos *et al*. reported that exogenous RANTES reduced angiogenesis in mice at day 14 in a disc-induced inflammatory angiogenesis assay^40^. Furthermore, Cochain *et al*. did not evidence any control of the RANTES-CCR5 axis on post-ischemic vessel growth^41^. In contrast, it was demonstrated that RANTES is required for angiogenesis following peripheral ischemia in a rat hindlimb ischemia model^42^. Moreover, Ambati *et al*. reported that CCR5-deficient mice experienced a sustained inhibition of corneal neovascularization after chemical and mechanical denudation of corneal epithelium^43^. We previously reported that RANTES may promote angiogenesis in a subcutaneous model of RANTES-base scaffold local implantation in rat^12^. Indeed, the incorporation of RANTES in a commercial nitrocellulose scaffold through non-covalent interactions resulted in a local angiogenesis after its subcutaneous implantation in rat^12^. In the present study, RANTES incorporated in pullulan/dextran microparticles (MP) was mostly localized on their surface, with some RANTES identified in the core of the MP. Since covalent chemical bonds between RANTES molecules and the polymers could lead to potential chemical modification and prevent the biological effect of RANTES, we selected another strategy to incorporate them into the MP. Freeze dried MP were rehydrated with a solution of RANTES, leading to the formation of a gel-like scaffold negatively charged able to retain the molecules within the hydrophilic polymer network. Our results demonstrated that RANTES could be released from MP for at least 14 days and that MP enzyme-induced biodegradation could provide an optimal angiogenic dose of RANTES^12,39–41^. We hypothesize that electrostatic interactions between positively charged residues in RANTES and the negatively charged phosphoester bonds of the scaffold might be responsible for the high affinity of RANTES for the MP and that a slow degradation preserves RANTES from homeostasis degradation. Indeed, pullulan-dextran cross-linking occurs through the formation of phosphoester linkages between two hydroxyl groups within the polymer chains^44^.

Increasing the concentration of charged polymers is an attractive approach to retain growth factors within hydrogel networks through restricted diffusion out of the networks^45^. A bio-inspired approach to increase the magnitude of these electrostatic interactions relies on animal-derived sulfated glycosaminoglycans such as chondroitin sulfate and heparan sulfate to obtain growth factors-binding biomaterials^45^. We recently demonstrated that a scaffold containing fucoidan, a sulfated marine polysaccharide, and VEGF, significantly decreased its release and guided the generation of functional vascular networks^25^. Similarly, we demonstrate that the effect of RANTES on revascularization and on muscular regeneration was notably improved when it was injected with MP, suggesting that the biomaterial may preserve the chemokine from proteolysis. Our study analyzed the RANTES-induced effects on angiogenesis and muscle regeneration at day 10 post-ischemia. A time point at day 5 post-ischemia would be helpful at allowing better delineations of association/causality. Moreover, perfusion imaging using a Laser Doppler imaging system would be helpful to measure the blood flow of the ischemic limb versus the control non-ischemic limb before and after surgery. Finally, our MP could be considered as tunable reservoirs through the number of phosphoesters linkages, since this number depends on the amount of cross-linker that is used during the MP preparation step.

The cooperation between inflammation and vessel formation represents an essential axis of the muscular regeneration process^46^. Recruited monocytes-macrophages secrete growth factors, cytokines and chemokines involved in myogenic cell recruitment^7^, and satellite cell proliferation and migration^47^. The RANTES-induced myogenic cell migration may participate in the tissue regeneration^48^. The expression of CCR5 and SDC-4 by myoblasts may reinforce the role of RANTES in the recruitment and the proliferation of myoblasts^49^.

The presence of functional RANTES is required for angiogenesis following peripheral ischemia in rats^42^. The positive effects of RANTES to induce neo-angiogenesis and muscle regeneration may be of interest to restore blood flow to ischemic tissues after vessel occlusion. In addition to its effect on endothelial cells, RANTES also induces the recruitment of smooth muscle progenitor cells^50^. The mobilization of EPC from bone marrow to the blood flow, their migration to the site of ischemia, and their incorporation into neo-vessels are essential steps of limb tissue repair process^51^. However, it is to note that in our study, very few CD34 + CD31 + vWF + cells could be observed. The difficulty resides in the low number of circulating EPC in the blood as well as that of recruited and incorporated EPC in the targeted vasculature.

Therefore, to assess the *in vivo* potential of RANTES-loaded MP to enhance the specific recruitment of EPC, future studies should be conducted based on the administration of RANTES-loaded MP combined with an intravenous injection of fluorescent-labeled EPC in Nude mice as described^20^. It was previously demonstrated that SDF-1/CXCL12 could regulate EPC differentiation to endothelial mature cells^33^. Other chemokines may exert such a pro-angiogenic effect: MCP-1/CCL2 is the first CC chemokine reported to play a direct role in tumor angiogenesis^52^, eotaxin also induces *in vivo* angiogenic responses by using endothelial cells expressing CCR3^53^ and numerous studies highlighted the role of CXCL4 in angiogenesis^54^. The reduced pro-angiogenic effects observed after cell treatment by [E66A]-RANTES as compared to RANTES highlighted a slight role of chemokine oligomerization. However, the relationship of RANTES with other chemokines with well-known angiogenic effects should also be taken into account. It has been described a CXCL4-CCL5 heterodimer formation, and a peptide inhibition of CXCL4-CCL5 interactions resulted in a reduced progression of human aortic aneurysm^55^.

In parallel, the injection of EPC may represent a therapeutic alternative to the local injection of pro-angiogenic growth factors. To improve the therapeutic effects of EPC delivered by intra-muscular or intra-arterial injection, the aim of actual studies is to develop adjunctive therapies, along with molecular and bioengineering tools^56^. As only 5–30% of microvessels originated from engrafted EPC^34^, the injection of both EPC and a pro-angiogenic factor, such as RANTES, may represent an innovative therapeutic strategy leading to an increased revascularization after hindlimb ischemia. RANTES-induced homing of EPC may represent a mechanism underlying angiogenesis. It was previously demonstrated that platelet aggregation releases chemokines such as RANTES. Platelets can enhance neovascularization at least partly by RANTES-enhanced CD34 + cell adhesion^57^. In addition to the role of RANTES in the induction and modulation of inflammation, RANTES stimulates the homing and recruitment of EPC in renal vascular regeneration^58^, in atherosclerotic plaque^59^ and in wound healing^60^. Our data also demonstrated that the binding of RANTES to glycosaminoglycans, as evidenced by [^44^AANA^47^]-RANTES, to GPCR, CCR5, or to proteoglycans, SDC-4 and CD44, present at the membrane of EPC, is essential to the RANTES-induced *in vitro* biological effects such as spreading, migration and vascular sprout formation. Nevertheless, the moderate decrease of cell migration towards RANTES mutants as compared to cell migration towards RANTES suggest that RANTES binding to glycosaminoglycans or RANTES oligomerization are not the only mechanisms involved in RANTES-induced cell migration. It would be of interest to better characterize the respective effect of CCR5, SDC-4 and CD44 expressed by EPC when these cells migrate towards RANTES mutant. It is conceivable that RANTES may interact with the protein core of SDC-4 or CD44. For this purpose, experiments based on neutralizing antibodies specific of SDC-4 or CD44 will be conducted.

The role of CCR5 will also be further investigated by siRNA interference or using specific neutralizing antibodies. It has previously been described that the [^44^AANA^47^]-RANTES mutant showed a 80-fold reduction in affinity for CCR1, despite normal binding to CCR5, and was able to induce monocyte chemotaxis at micromolar concentrations, conversely to nanomolar concentrations for native RANTES^61^. The lower chemotactic effect induced by [E66A]-RANTES compared to [^44^AANA^47^]-RANTES may be related to the use of a positively charged and fully exposed motif, KKWVR, of CCL5 oligomer in GAG binding^62^. Moreover, future studies will be carried out to assess the putative effect of RANTES on human EPC survival, proliferation and differentiation potential into mature human endothelial cells. Moreover, the lower biological effects induced *in vitro* by [^44^AANA^47^]-RANTES encourage us to deliver it *in vivo* to verify its lower capacity compared to RANTES to induce revascularization and muscle regeneration after mice hindlimb ischemia induction.

Altogether, our results highlight the therapeutic role of RANTES-loaded MP after hindlimb ischemia that is involved in the chemoattraction of inflammatory cells, of mature endothelial cells and of endothelial progenitor cells, leading to the revascularization and tissue regeneration. Therefore, future studies based on the injection of biomaterials containing both human EPC and RANTES in order to deliver them locally by intra-muscular injection at the site of ischemia may represent a putative innovative therapeutic approach to counteract hindlimb ischemia and to avoid an invasive surgery.

## Methods

### Materials

RANTES and VEGF were purchased from R&D Systems (Lille, France). RANTES biotinylated at residue 1 (B-RANTES), [E66A]-RANTES and [^44^AANA^47^]-RANTES were synthesized by L. Martin and C. Vita (CEA Saclay, Gif-sur-Yvette, France) as described^11^. [E66A]-RANTES leads to a disaggregated RANTES with impaired ability to oligomerize^60^ and [^44^AANA^47^]-RANTES, mutated in the main GAG binding domain, exhibits a potent anti-inflammatory effect^63–65^.

### Microparticle preparation

Microparticles (MP) were obtained using water-in-oil (w/o) emulsification process^66^. Briefly, 75% pullulan (Mw 200000, Hayashibara Inc., Japan) and 25% dextran (Mw 500000, Sigma-Aldrich, Lyon, France) were dissolved in water then dispersed in canola oil under mechanical stirring^66^. Polysaccharides were cross-linked by sodium trimetaphosphate at 50 °C for 20 min. Resulting MP were washed with PBS then sieved using a vibrating shaker (AS 200, Retsch, Eragny sur Oise, France) to obtain particles of 300–500 µm diameter. MP were freeze-dried and stored at room temperature. For experiments, MP were suspended in PBS (15 mg/mL). The association of RANTES (10 nM) to the MP for 2 hours at 37 °C was verified by the use of biotinylated RANTES revealed with avidin-FITC (DAKO, Les Ulis, France) by confocal microscopy (Leica SP8 tandem, Wetzlar, Germany).

The resorption of RANTES-loaded MP was assessed after MP incubation with RANTES (10 nM) for 2 hours at 37 °C. Then MP were transferred to PBS and incubated for 30 minutes, 24 hours, 7 days and 14 days at 37 °C. At day 14, MP were incubated with a pullulanase/dextranase solution to simulate their bio-resorption (1:1 v/v) (Sigma Aldrich). The release profile of RANTES from MP was assessed by ELISA assay (R&D system, Lille, France).

### Mouse model of hindlimb ischemia

The animal protocol was approved by the Bichat Hospital Institutional Animal Care and Use Committee and all experiments conformed to European Community guidelines for the care and use of laboratory animals. Six week-old male white BALB/c mice, weighting 20–25 g (Janvier, CERJ, Laval, France), were anesthetized with intraperitoneal ketamine (10%, 8 mg/kg) and xylazine (5%, 8 mg/kg) solution (Bayer, Puteaux, France). BALB/c mice represent a good model for hindlimb ischemia development^67^ and the sample size (n = 5 mice per group) was calculated for the study according to the objective and design of the study, literature analysis and statistical test based on acceptable level of significance; power of the study, expected effect size, underlying event rate, standard deviation^68^. The right *profunda femoris* artery was ligated to induce a hindlimb ischemia whereas the left muscle was kept healthy. Ten minutes later, treatments were administered by an intramuscular injection of 20 µL in the central part of ischemic limb at a distance equivalent to the middle of the distance between the two articulations. Thirty mice were divided into six groups (with n = 5 mice per group) according to the treatment injected: a) 10 nM RANTES-loaded MP (300 µg of MP at 15 µg/µL in PBS), b) 2 nM VEGF-loaded MP, c) MP incubated with PBS, d) 10 nM RANTES solution, e) 2 nM VEGF solution, f) PBS solution. Mice were euthanized by a pentobarbital injection at day 10 after the surgery.

The ischemic and non-ischemic (control) gastrocnemius muscles were collected, frozen in isopentane solution cooled in liquid nitrogen and stored at −80 °C for histological analysis.

### Clinical score of muscle regeneration

Mice behavior was recorded at day 0, 5 and 10 after the induction of hindlimb ischemia. At day 0, the observation was performed immediately after hindlimb ischemia. At each time point, a clinical score was calculated for each mice group as the sum of the grade obtained for three different scales referring to completely different parameters: the Tarlov scale^69^, a mouse limb ischemia grading scale^70^, and a modified ischemia scale to detect less severe levels of ischemia^71^, to evaluate the functional grading of muscle regeneration (Table 1).

**Table 1:** Clinical score parameters.

Tarlov score function	
0	No movement
1	Barely perception movement, no weight bearing
2	Frequent and vigorous movement, no weight bearing
3	Supports weight, partial weightbearing
4	Walk with mild deficit
5	Normal, slow walking
6	Full and fast walking
**Ischemia score tissue grade**	
0	Auto amputation, half lower limb
1	Gangrenous tissue, half foot
2	Gangrenous tissue grade, half foot with lower limb muscle necrosis
3	Gangrenous tissue grade, half foot without lower limb muscle necrosis
4	Pale foot or gait abnormalities
5	Normal
**Modified ischemia score tissue grade**	
0	Autoamputation of leg
1	Leg necrosis
2	Foot necrosis
3	Two or more toe discoloration
4	One toe discoloration
5	Two or more nail discoloration
6	One nail discoloration
7	No necrosis

Tarlov score function, ischemia score tissue grade, modified ischemia tissue grade.

### Histology and immunohistochemistry

Frozen 7 µm-thick sections of ischemic gastrocnemius muscle were fixed in acetone and stained with hematoxylin-eosin (H&E). Images were obtained by digital slide scanner Nanozoomer 2.0RS (Hamamatsu, Massy, France) and analyzed with NDPI (Hamamatsu, Massy, France).

The number per field of neo-formed capillaries was counted on H&E sections. To localize biotinylated RANTES at 5 and 10 days, muscle tissue cryosections were revealed with streptavidin-FITC labeling and with an antibody anti-RANTES (10 µg/mL, polyclonal goat IgG, R&D System) revealed with a secondary antibody coupled to HRP then with DAB and counterstained with hemalun.

To assess muscle regeneration, sections were immunostained with an anti-myogenin D antibody (1/100, rabbit polyclonal IgG, Abcam, Paris, France) overnight at 4 °C, revealed by an Alexa Fluor 488 conjugated secondary antibody (1/500, Molecular Probes, Invitrogen, France).

Muscle regeneration was expressed as the ratio of myogenin D positive cells to 4,6 diamidino-2-phenylindole hydrochloride (DAPI)-stained cells. Sections were also incubated with DAPI (1 mg/mL) solution (Sigma-Aldrich). Inflammatory cell infiltration was evidenced with an antibody directed against monocytes-macrophages (MOMA-2, 1/100, rat IgG2b, Abcam, France)^64^, overnight at 4 °C, then revealed with biotinylated IgG antibody and 3,3-Diaminobenzidine (DAB, DAKO, France). Slides were counterstained in hemalum (Sigma-Aldrich). Tissue areas were measured with ImageJ software. The results were expressed as the ratio of the infiltrated cells surface area to the total surface area of the section as previously described^72,73^. Vessels were revealed with anti-endothelial cell marker CD31 (1/100, rat monoclonal IgG1, Abcam), anti-alpha smooth muscle actin (SMA; 1/200, DAKO), anti-CD34 (1/100, mouse monoclonal IgG1, Cell Signaling, Leiden, The Netherlands) or anti-von Willebrandt factor (vWf) antibodies (1/100, DAKO). All images were obtained by digital slide scanner Nanozoomer 2.0RS (Hamamatsu, Massy, France) and analyzed with NDPI software (Hamamatsu, Massy, France).

### Cell culture

Human endothelial progenitor cells (EPC, number BSP 284, provided by J. Larghero, Hospital St Louis, Paris, France) as described^74^ and cultured in EGM-2 MV (Lonza, Levallois-Perret, France) supplemented with foetal bovine serum 5%, EGF (Epidermal Growth Factor, 5.0 ng/mL), Hydrocortisone (0.2 μg/mL), VEGF (Vascular Endothelial Growth Factor, 0.5 ng/mL), bFGF (basic Fibroblast Growth Factor, 10 ng/mL), R3 IGF-1 (Insulin like Growth Factor, 20 ng/mL), Ascorbic Acid (1 μg/mL), antibiotics (penicillin- streptomycin, 1%, Invitrogen, Cergy-Pontoise, France) and L-Glutamine (1%, Invitrogen). The medium was changed twice a week.

### Flow cytometry analysis

To identify the expression of heparan sulfate chains and chemokine receptors on EPC membranes, cells were incubated for 1 h at 4 °C with anti-CCR1, anti-CCR5, anti-CCR3, anti- SDC-1, anti-SDC-4, anti-CD44 or anti-heparan sulfate (HS) antibodies or their isotype (10 µg/mL). After washing, cells were labeled either with Alexa Fluor 488 goat anti-mouse IgG or IgM antibodies (1/1000, Invitrogen, France). Binding of biotinylated RANTES (B- RANTES) at 20 nM or 40 nM on EPC membrane, was revealed by avidin-Alexa Fluor 488 complex (R&D system). Cells were fixed in 1% paraformaldehyde (PFA) (Sigma-Aldrich) and analysed on a FACScan (Becton Dickinson, Le Pont-de-Claix, France).

### Spreading assay

After a 24 h serum deprivation, 20 × 10^3^EPC per well were seeded on a fibronectin (100 μg/mL, BD Biosciences Pharmingen, Le Pont de Claix, France) pre-coated 8-well Labtek and incubated for 2 h at 37 °C with 3 nM RANTES to assess their spreading. In a parallel experiment, EPC were pre-incubated for 2 h at 37 °C with anti-CCR5, anti-CD44, or anti- SDC-4 antibodies or their isotypes (5 µg/mL). Alternatively, cells were preincubated with beta-D-xyloside (1 mM) for 72 h, or RANTES was pre-incubated with low molecular weight heparin (10 µg/mL, Sigma-Aldrich) for 2 h, as previously described^12^. Cells were permeabilized in 0.05% Triton X-100 (Sigma-Aldrich), fixed with PFA (1%) then stained with AlexaFluor 568 phalloidin (Molecular Probes, Invitrogen) and filamentous actin was observed with a fluorescence microscope (Zeiss, AXIOPHOT, N°/MicMac, France S.A). Ten fields of stained cells were photographed for each treatment. Cell areas expressed in square inches were evaluated on 30 cells by treatment with the Scion Imager software (Scion Image Software and National Institutes of Health, Release Beta 3b Software).

### Cell migration assays in Boyden chambers

The RANTES-induced chemotactic effect was assessed by placing EPC in the upper part of a modified Boyden chamber coated with fibronectin (100 µg/mL, BD Biosciences Pharmingen, France) and 3 nM RANTES deposited in the lower chamber, alone or preincubated with heparin (10 µg/mL, Sigma-Aldrich) for 2 h. Alternatively, EPC were chemoattracted towards [^44^AANA^47^]-RANTES or [E66A]-RANTES (each at 3 nM). In parallel, EPC were pre-incubated for 2 h at 37 °C with anti-CCR5, anti-CD44, anti-SDC-4 antibodies or their respective isotypes (5 µg/mL). The involvement of GAGs on RANTES-induced effects was assessed after a 72 h cell incubation with beta-D-xyloside. Cells were fixed, stained with Mayer’s hemalum (Sigma-Aldrich), observed with a phase contrast microscope (Nikon Coolpix 8400, Nikon Corporation, Tokyo, Japan, × 20 objective) and counted by two blinded-observers.

### *In vitro* angiogenesis assay

2D-angiogenesis assay was performed with 4 × 10^4^ EPC per well seeded on Matrigel-coated 8-well Labtek and incubated for 16 h with 3 nM RANTES, preincubated or not with heparin (10 µg/mL), or with [^44^AANA^47^]-RANTES or [E66A]-RANTES. Alternatively, EPC were pre-treated for 2 h either with anti-CCR1, anti-CCR5, anti-SDC-1, anti-SDC-4, anti-CD44, or with their respective isotypes (5 µg/mL), or with beta-D-xyloside (1 mM) for 72 h. After 16 h, vascular tubes were fixed with 4% PFA, stained with 0.05% Cristal violet (Sigma- aldrich) and photographed with phase contrast microscopy (OLYMPUS CK40). The average length (in µm) and area (in µm^2^) of 40 vascular tubes were evaluated using the Scion Imager System (Scion Image Software and National Institutes of Health, Release Beta 3b Software).

### Statistical Analysis

Results are expressed as mean values ± SEM for three different experiments. An ANOVA test followed by a Fisher’s exact test was performed with Statview software (StatView 4.5 Abacus Concepts, Berkeley, USA). A *P* value < 0.05 was used as the criterion of statistical significance.

### Ethical approval

All applicable institutional guidelines for the care and use of animals were followed. All procedures performed in studies involving animals were in accordance with the ethical standards of the institution or practice at which the studies were conducted. This article does not contain any studies with human participants performed by any of the authors.

## Electronic supplementary material

Supplementary figure 1
