# Supplementary figures and images for: Pro-angiogenic effect of RANTES-loaded polysaccharide-based microparticles for a mouse ischemia therapy
Source: Sci Rep. 2017 Oct 16;7:13294. doi: 10.1038/s41598-017-13444-7 (PMC5643514; doi:10.1038/s41598-017-13444-7)

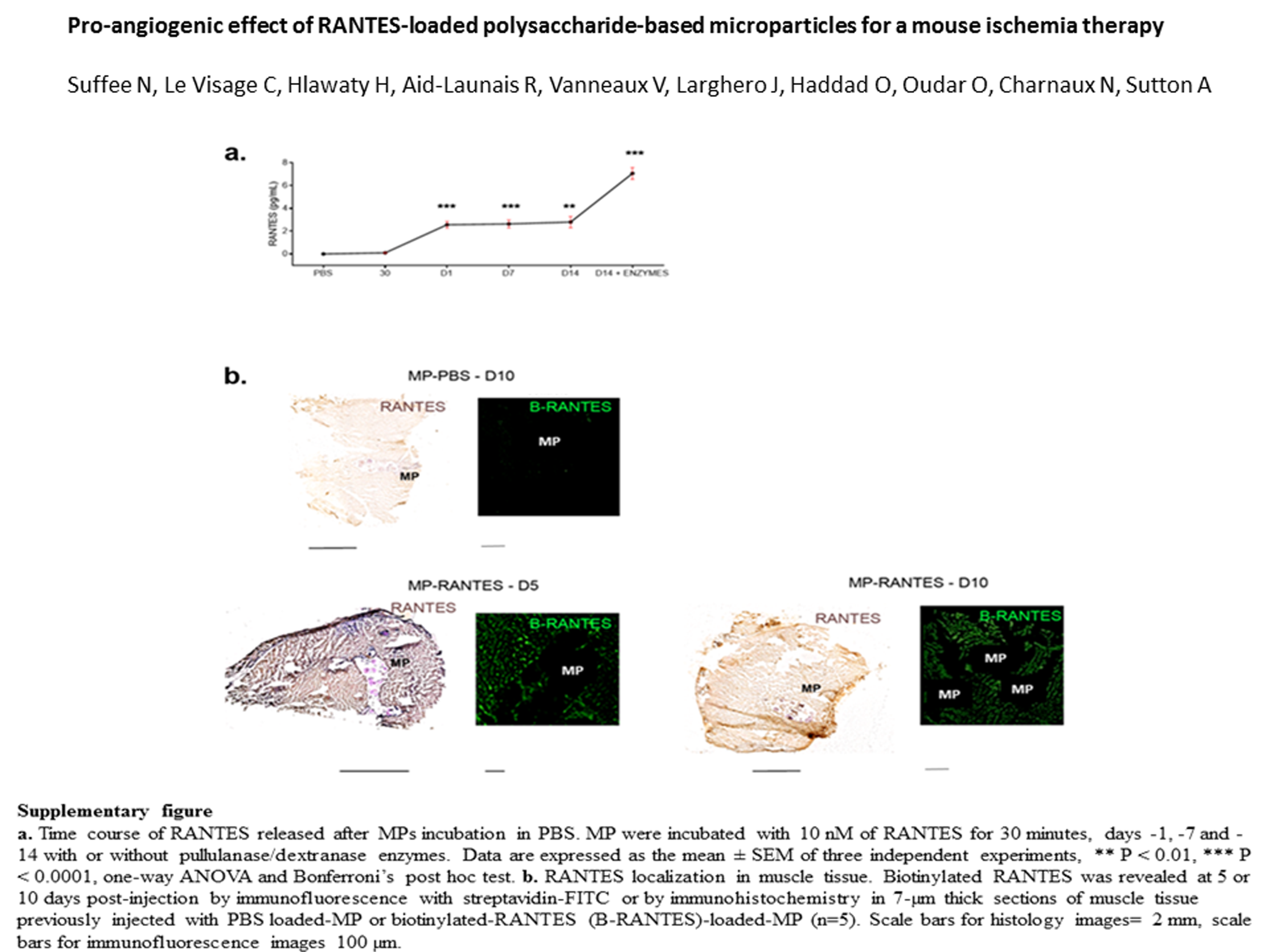

Supplement: Supplementary file 1 — Supplementary figure 1 [file 41598_2017_13444_MOESM1_ESM.tif]
